# Developmental Exposure to Endocrine Disrupting Chemicals and Its Impact on Cardio-Metabolic-Renal Health

**DOI:** 10.3389/ftox.2021.663372

**Published:** 2021-07-05

**Authors:** Radha Dutt Singh, Kavita Koshta, Ratnakar Tiwari, Hafizurrahman Khan, Vineeta Sharma, Vikas Srivastava

**Affiliations:** ^1^Department of Physiology and Pharmacology, University of Calgary, Calgary, AB, Canada; ^2^Systems Toxicology and Health Risk Assessment Group, Council of Scientific and Industrial Research-Indian Institute of Toxicology Research, Lucknow, India; ^3^Academy of Scientific and Innovative Research, New Delhi, India; ^4^Feinberg Cardiovascular and Renal Research Institute, Feinberg School of Medicine, Northwestern University Chicago, Chicago, IL, United States

**Keywords:** prenatal exposure, DOHAD, cardio-metabolic-renal health, endocrine disrupting chemicals, cardiovascular disorder

## Abstract

Developmental origin of health and disease postulates that the footprints of early life exposure are followed as an endowment of risk for adult diseases. Epidemiological and experimental evidence suggest that an adverse fetal environment can affect the health of offspring throughout their lifetime. Exposure to endocrine disrupting chemicals (EDCs) during fetal development can affect the hormone system homeostasis, resulting in a broad spectrum of adverse health outcomes. In the present review, we have described the effect of prenatal EDCs exposure on cardio-metabolic-renal health, using the available epidemiological and experimental evidence. We also discuss the potential mechanisms of their action, which include epigenetic changes, hormonal imprinting, loss of energy homeostasis, and metabolic perturbations. The effect of prenatal EDCs exposure on cardio-metabolic-renal health, which is a complex condition of an altered biological landscape, can be further examined in the case of other environmental stressors with a similar mode of action.

## Introduction

Environmental toxicants comprise a wide range of chemical agents released through natural or anthropogenic sources. They contaminate the abiotic components of the ecosystem and affect the health of the biotic components (Gore et al., 2015; Trasande et al., 2016). Endocrine disrupting chemicals (EDCs) include phenols, phthalates, parabens, flame retardants, heavy metals, pesticides, perfluorinated chemicals, UV filter components, triclosan, and organochlorines. Among these, of particular concern are polychlorinated biphenyls (PCBs), polybrominated biphenyls (PBBs), dioxins, bisphenols, dichlorodiphenyltrichloroethane (DDT), vinclozolin, diethylstilbestrol (DES), and heavy metals, such as cadmium, mercury, arsenic, lead, manganese, and zinc. On a daily basis, people could be exposed to EDCs through packaged foods, plastics, cosmetics, and pharmaceuticals in multiple personal and occupational settings (Tchounwou et al., 2012; Gore et al., 2015; Marcoccia et al., 2017). Cumulative exposure to mixtures of EDCs can lead to adverse effects on the health of the exposed individuals (Crews et al., 2003). Multiple studies, including the studies of the National Health and Nutrition Examination Survey (NHANES), have shown that about 75–97% of US and Asian adults have detectable levels of phthalates and phenols [bisphenol A (BPA) and polyfluoroalkyl chemicals] in their urine (Silva et al., 2004; Calafat et al., 2007, 2008; Vandenberg et al., 2010; Zhang et al., 2011; Husøy et al., 2019). Epidemiological and experimental studies have also linked adult exposure to EDCs with abnormal male and female reproductive health, diabetes, obesity, cardiovascular and metabolic disorders, thyroid function, and hormone sensitive cancers (Howard and Lee, 2012; Bodin et al., 2015; Heindel et al., 2015, 2017). Children are also vulnerable to EDCs (Calafat et al., 2017; Hendryx and Luo, 2018), making EDC exposure a major health concern for all age groups.

While adult exposure to EDCs has been proved to promote adverse health effects, the developing fetus could have greater susceptibility due to its higher rate of growth and cellular differentiation (Barouki et al., 2012; Grandjean et al., 2015). Adverse health effects can be highly pronounced in the developing fetus at concentrations of EDCs much lower than the permissible limits (Welshons et al., 2003). Multiple epidemiological studies provide evidence that exposure to EDCs in pregnant women is nearly universal (Woodruff et al., 2011; Arbuckle et al., 2015; Lee et al., 2017; Rosofsky et al., 2017; Philips et al., 2018). Gestational exposure to EDCs may occur by way of daily care products, such as cosmetics, the use of electronic devices, and consumption of the animal, plant, or processed foods (Caserta et al., 2011; Rouillon et al., 2017).

Apart from gestation, childhood and adolescence are also highly vulnerable periods. The kidney and liver of infants are immature and have relatively poor glomerular filtration and capacity to detoxify drugs and chemicals (Seely, 2017). A growing body of evidence suggests that *in utero* exposure to EDCs, such as BPA, phthalates, polyfluoroalkyl chemicals, and heavy metals, is associated with preterm birth and fetal growth restrictions (Wolff et al., 2008; Govarts et al., 2012; Bach et al., 2015; Veiga-Lopez et al., 2015; Birks et al., 2016; Lenters et al., 2016; Lauritzen et al., 2017). These two conditions are known risk factors for the early onset of renal, cardiovascular, and metabolic dysfunction (Woodruff et al., 2011; Barker, 2012; Mierzynski et al., 2016; Martin et al., 2017).

In the present review, the effects of prenatal, perinatal, and early-life exposure to EDCs on the cardio-metabolic-renal health have been described based on epidemiological and experimental evidence available. The common and unifying mechanisms, which may be involved across multiple EDCs, are also discussed.

## Prenatal exposure to EDCS and Metabolic Syndrome

Metabolic syndrome can be defined as the state of metabolic perturbation, which includes at least three of the following five symptoms: elevated waist circumference, elevated triglycerides, reduced high-density lipoprotein cholesterol (HDL-C), elevated fasting glucose, and hypertension (Alberti et al., 2009). Metabolic syndrome (MetS) was reported to be associated with an increased risk of chronic diseases, including type 2 diabetes (T2D), non-alcoholic fatty liver disease (NAFLD), various cancer forms, and cardiovascular diseases (Mendrick et al., 2018). As the endocrine system homeostasis is crucial for normal development and metabolism, the endocrine disrupting chemicals can also be referred to as “metabolism disrupting chemicals.” The link between developmental exposure to endocrine disruptors and the onset of metabolic perturbations was pioneered by Grun and Blumberg who described the obesogenic action of organotins (Grün and Blumberg, 2006).

The epidemiological studies on maternal exposure to EDCs and the risk of metabolic disorders have been summarized in Table 1. The experimental studies on this are summarized in Table 2. Prenatal exposure to BPA causes hyperleptinemia, high blood pressure, and enhanced weight gain during early childhood (Ashley-Martin et al., 2014; Bae et al., 2017; Guo et al., 2020). Metabolic disruption after maternal BPA exposure has been reported in rodent models where it promoted hyperlipidemia, altered glucose homeostasis, impaired energy expenditure, and increased adiposity (Miyawaki et al., 2007; Alonso-Magdalena et al., 2010; MacKay et al., 2013; Li et al., 2014; García-Arévalo et al., 2016; Bansal et al., 2017; Desai et al., 2018b; Manukyan et al., 2019; Diamante et al., 2020).

**Table 1:** Epidemiological studies on maternal exposure to EDCs and risk of metabolic disorders.

**EDC**	**Study area**	**Key findings**	**References**
BPA	Maternal-Infant Research on Environmental Chemicals Study (MIREC) (*n =* 1,363)	Hyperleptinemia in females but lower serum adiponectin levels	Ashley-Martin et al., 2014
	Rural area of East China cohort study (*n =* 403)	Increase in BMI and waist to height ratio at 7 year of age	Guo et al., 2020
	Birth cohort study (*n =* 645)	Higher diastolic BP while systolic BP did not differ significantly	Bae et al., 2017
Diethylstilbestrol (DES)	National Cancer Institute DES Follow-Up Study (*n =* 2,871)	Increase in body mass index, weight gain, waist circumference, and adult obesity in females	Hatch et al., 2015
Phthalates	New York, USA. Maternal urine (3rd trimester of pregnancy) (*n* = 173)	Lower fat mass and BMI in children exposed to di(2- ethylhexyl) phthalate (DEHP)	Buckley et al., 2016a
	Maternal-Infant Research on Environmental Chemicals Study (MIREC) (*n =* 1,363)	Increased odds of high leptin among males along with increase in maternal Mono-(3-carboxypropyl) (MCPP)	Ashley-Martin et al., 2014
Persistent organic pollutants (POPs)	Spanish birth cohort study (*n =* 2,483), 1,1-Dichloro-2,2-bis(p-chlorophenyl) ethylene (DDE)	24% rapid growers and 30% overweight infants in DDE exposed group	Valvi et al., 2014
	Faroe Islands (*n =* 561), Polychlorinated biphenyls (PCBs) and DDE	Higher odds of overweight in 5–7 years old children with increase in PCBs and DDE in maternal serum	Tang-Péronard et al., 2014
	Prospective Faroese Birth Cohort study (*n =* 656), PCBs and DDE	Hyperinsulinemia in female offspring at early childhood along with increase in maternal POP levels	Tang-Péronard et al., 2015
Heavy metals	The Newborn Epigenetics Study (NEST) (*n =* 319), Cadmium	Increased risk of juvenile obesity	Green et al., 2018
	Maternal–Infant Research on Environmental Chemicals (MIREC) study, Canada (*n =* 2001), Cadmium	Increased odds of hyperleptinemia in male offspring	Ashley-Martin et al., 2015
	Human Early Life Exposome (HELIX) project (*n =* 805), Mercury	Increased odds of metabolic syndrome in children	Stratakis et al., 2020
	Maternal Mercury levels 2.11 (1.04–3.70) μg/L	Increased odds of overweight and obesity in children aged 2–15 years, however plenty of maternal folate intake was associated with a 34% reduction in obesity risk after Hg exposure	Wang et al., 2019
	Strong Heart Family Study (*n =* 466), Arsenic	Increased odds of offspring with Type 2 Diabetes	Tinkelman et al., 2020
	Early Life Exposure in Mexico to Environmental Toxicants (ELEMENT) birth cohort study, Mexico (*n =* 369), Lead Mean Lead concentration was 43 μg/dL	Significantly lower total cholesterol level in males (Pb > 5 μg/dL) No association in female offspring	Liu et al., 2020

**Table 2:** Major experimental studies describing maternal exposure to EDCs and risk of metabolic disorders in offspring.

**EDC class**	**Model and dose**	**Key findings**	**References**
Bisphenol A (BPA)	Mice (C57BL/6J) (BPA 10 μg/kg/day to 10 mg/kg/day), WPM2 to PND28	Impaired glucose stimulated insulin secretion (GSIS), reduced β-cell mass and pancreatic islets inflammation only in males	Bansal et al., 2017
	Rats (SD) (BPA 40 μg/kg/day), GD0 to PND28	Glucose intolerance, downregulated expression of glucokinase (Gck) gene in liver	Li et al., 2014
	Mice (ICR) (BPA 1–10 μg/kg/day + High fat diet), GD10 to PND31	Increased BMI, increase in serum TG and cholesterol	Miyawaki et al., 2007
	Mice (OF-1) (BPA 10–100 μg/kg/day), GD9-GD16	Glucose intolerance, elevated plasma triglycerides (TG) and insulin levels, hyperleptinemia, no change in BMI	Alonso-Magdalena et al., 2010
	Mice (C57BL/6J) (BPA 5 μg/kg/day), GD0 to GD21	Decrease in liver TG levels in females	Diamante et al., 2020
	Rats (Fischer 344) (BPA 0.5 or 50 μg/kg/day) GD3.5 to GD22	Reduced glucose stimulated insulin secretion	Manukyan et al., 2019
	Mice (OF-1) (BPA 10 and 100 μg/kg/day), GD9 to GD16	Elevated levels of plasma insulin and leptin. Reduced pancreatic β-cell mass in males	García-Arévalo et al., 2016
	Rats (SD) (BPA 5 mg/L), WPM2 to PND28	Increased adiposity, lipid content, upregulated expression of appetite peptide	Desai et al., 2018a,b
	Mice (CD-1) (BPA 5 to 50,000 μg/kgday), GD9 to GD18	Increase in body weight, abdominal adipose tissue mass, serum leptin and insulin levels and glucose intolerance	Angle et al., 2013
	Mice (CD-1) (BPA 3.49–7.2 μg/kg/day through diet), GD0 to PND28	Glucose intolerance in only males, HFD exacerbated obesogenic effect in females	MacKay et al., 2013
Phthalates	Rats (Wistar) (DEHP 1, 10 and 100 mg/kg/day), GD9 to GD21	Hyperglycemia, hyperinsulinemia at PND60, DNA methylation in β- cell development genes	Rajesh and Balasubramanian, 2015
	Mice (DEHP 0.2, 2, 20 mg/kg/day) WPM1 to GD21	Elevated adipogenesis, glucose intolerance, hypertrophic adipocytes, and dysbiosis of gut microbiota	Fan et al., 2020
	Rats (SD) (DEHP 600 mg/kg) throughout pregnancy and lactation	Decrease in serum alanine transaminase (ALT), total protein (TP), blood urea nitrogen (BUN), and creatinine, and elevated thyroid stimulating hormone (TSH) levels	Zhang et al., 2018
	Mice (CD-1) (DEHP 20, 200 μg, 500 or 750 mg/kg/day), GD10.5 to GD21	Mild liver damage, immune cells infiltration and altered DNA Methylation in liver	Wen et al., 2020
	Mice (PCNA^Y114F^/ ^Y114F^) (DEHP 0.05, 500 mg/kg/day), WPM3 to PND21	Upregulated phosphorylation of PCNA at Y114 Enhanced adipocyte differentiation, enhanced body weight gain	Hunt et al., 2017
	Mice (C57BL/6) (DEHP 30 mg/kg) throughout pregnancy and lactation.	Hypertrophic adipocytes, elevated serum cholesterol levels, elevated blood pressure	Lee et al., 2016
Diethystilbestrol (DES)	Rats (Wistar) (0.1 ppm DES), WPM2 to PND22	Prolonged gestational period, decrease in body weight only in females	Cagen et al., 1999b
DDT	Rats (SD) (DDT 25, 50 mg/kg /day), GD8 to GD14	50% of F3 male and female were obese	Skinner et al., 2013
	Mice (C57BL/6J) (DDT 1.7 mg /kg/day), GD11.5 to PND5	Impaired cold tolerance, increased body fat content in females. HFD exaggerated glucose intolerance and dyslipidemia	La Merrill et al., 2014
Persistent organic pollutants (POPs)	Mice (C57BL/6JxFVB hybrid) (TCDD 10– 10,000 pg/kg/day; PCB 153 0.09–1,406 μg/kg/day) through feed during gestation and lactation	TCDD exposed females show increased fat content. PCB exposed males show hyperglycemia	Van Esterik et al., 2015
Heavy metals	Rats (CdCl_2_ 50, 500 μg/kg/day), WPM3 to PND21.	Glucose intolerance, increased insulin pancreatic secretion, increased circulating free fatty acids (FFAs)	Jacquet et al., 2019
	Zebrafish embryos (CdCl2 60 μg/L), from 4 to 7 dpf	Increased lipid accumulation at puberty	Green et al., 2018
	Mice (CD1)(CdCl_2_ and CH_3_HgCl 2 mg/kg bw), for 4 days before and 4 days after mating	Glucose intolerance, increased body weight and abdominal adiposity in males	Camsari et al., 2019
	Rats (Wistar) (10 ppm of CdCl_2_ *ad libitum*), weaning to mating and delivery	Hypermethylation of CpG on glucocorticoid receptor	Castillo et al., 2012
	Mice (CD-1) (CdCl2 500 ppb), GD0 to PND10	Increased body weight gain, mitochondrial dysfunction, disruption of retinoic acid and insulin signaling in females only	Jackson et al., 2020
	Mice (CD-1) (NaAsO_2_ 10 μg/L), GD10 to birth	Enhanced body weight gain, elevated body fat content, and hyperleptinemia	Rodriguez et al., 2016
	Mice (Swiss Webster) (100 μg/L NaAsO_2_), GD5 to GD21	Glucose intolerance, elevated fatty liver disease risk after HFD feeding	Ditzel et al., 2016
	Mice (C57BL/6J) (NaAsO_2_ 0, 100, and 1,000 μg /L) before mating to birth	Elevated fasting glucose levels, insulin resistance, high body weight in male offspring	Huang et al., 2018

*WPM, Week prior to mating; PND, Postnatal day; GD, Gestation day; TCDD:2,3,7,8-Tetrachlorodibenzo-p-dioxin; HFD, high fat diet; BW, body weight; hpf, hour post fertilization; dpf, days post fertilization*.

Pthalates are plasticizers generally used in many personal care and medical use products. Maternal exposure to phthalates was reported to cause metabolic disruptions, elevate body mass index (BMI), and leptin levels in the exposed population (Ashley-Martin et al., 2014; Buckley et al., 2016a,b; Maresca et al., 2016). Experimental studies have reported multiple mechanisms for phthalates-associated metabolic disruption, including damage and changes to hepatic metabolism (Zhang et al., 2018; Wen et al., 2020), enhanced adipose differentiation (Hunt et al., 2017), alteration in genes associated with β-cell developmental (Rajesh and Balasubramanian, 2015), and dysbiosis of gut microbiota (Fan et al., 2020). Altered phosphorylation of endothelial nitric oxide synthase and induction of angiotensin type 1 receptor could elevate blood pressure (Lee et al., 2016) and further contribute to metabolic disruption by phthalates.

Diethylstilbestrol is a non-steroidal chemical with estrogenic activity. It has been reported to act as an obesogen and cause metabolic disruption through enhanced weight gain in prenatally exposed human females (Hatch et al., 2015). However, in rodent models, gestational low dose (0.1 ppm) exposure to diethylstilbestrol (DES) resulted in reduced litter size and decreased body weight of the offspring (Cagen et al., 1999a,b).

Organochlorines, a different class of endocrine disruptors, have also been reported to cause metabolic deregulation after maternal exposure (Tables 1, 2). Human cohort studies on maternal exposure to dichlorodiphenyl-dichloroethylene (DDE) and PCBs showed an increase in the growth rate and childhood obesity in offspring (Tang-Péronard et al., 2014; Valvi et al., 2014). High maternal urinary DDE levels were also associated with increased body weight in infants (Karmaus et al., 2009; Valvi et al., 2014; Iszatt et al., 2015). Hyperinsulinemia has also been reported in some 5 year-old females exposed to persistent organic pollutants (Tang-Péronard et al., 2015). Experimental studies on prenatal exposure to DDE have also reported impaired cold tolerance, high body fat content, and the carryover of an obese phenotype up to F3 generation (Skinner et al., 2013; La Merrill et al., 2014). Female offspring, which were perinatally exposed to 2,3,7,8-Tetrachlorodibenzodioxin (TCDD), had increased fat while exposed males showed decreased fat content. In the same study, exposure to PCB was associated with increased glucagon levels in females, while males showed hyperglycemia (Van Esterik et al., 2015).

Many heavy metals, including cadmium, lead, mercury, and arsenic, are also reported to act through endocrine disruption at relatively low doses. In human birth cohort studies, cadmium and lead exposure during early lifetime resulted in juvenile obesity and hyperleptinemia (Ashley-Martin et al., 2015; Green et al., 2018). However, lower blood cholesterol levels were observed in male children after maternal lead (lead > 5 μg/dL) exposure (Liu et al., 2019, 2020). Maternal cadmium levels were also associated with juvenile obesity in the offspring (Green et al., 2018). Gestational exposure to cadmium has been reported to promote glucose intolerance, pancreatic damage, liver steatosis, and adiposity in the animal offspring up to F2 generation through endocrine disruption of glucocorticoid (Castillo et al., 2012) and retinoic acid signaling (Jackson et al., 2020). Arsenic exposure to both the mother and the offspring was associated with metabolic disorders, including T2D in the Strong Heart Family Study (Tinkelman et al., 2020). Multiple studies have reported metabolic disruption after gestational arsenic exposure, including increased glucose intolerance, adiposity, and insulin resistance (Rodriguez et al., 2016; Huang et al., 2018). High-fat diet has been found to exaggerate liver steatosis after maternal arsenic exposure in offspring (Ditzel et al., 2016). Cumulatively, all the above studies provide evidence that prenatal and early-life exposure to EDCs can cause metabolic dysfunction.

## Prenatal Exposure to EDCS and Chronic Kidney Disease

Chronic kidney disease is a growing health problem among children and adults. The incidence and the prevalence of chronic kidney disease (CKD) among children have been steadily increasing since the 1980s (Baum, 2010; Harambat et al., 2012; Becherucci et al., 2016). A number of traditional risk factors associated with CKD in children include hypertension, obesity, diabetes, and aberrant divalent mineral metabolism (Wong et al., 2006; Staples et al., 2010; Harambat et al., 2012; Warady et al., 2015). There is growing evidence that links exposure to EDCs with early progression to end-stage renal disease (ESRD) (Kataria et al., 2015). However, early-life exposure to EDCs and their association with chronic kidney disease have not been extensively studied. Some of the studies on gestational and early-life exposure to EDCs and their effect on the kidney are summarized in Table 3. Early-life exposure to EDCs was associated with elevated levels of kidney toxicity markers such as albumin-to-creatinine ratio (ACR), estimated glomerular filtration rate (eGFR), and urinary protein-to-creatinine ratio (UPCR) in some human population studies (Li et al., 2012; Trasande et al., 2013a, 2014; Malits et al., 2018).

**Table 3:** EDC exposure and renal function.

**EDC**	**Study system**	**Key findings**	**Reference**
Bisphenol A (BPA)	Cohort study of pediatric CKD patients from the US and Canada, BPA and phthalates	Increased tubular injury and oxidative stress	Jacobson et al., 2020
	National Health and Nutrition Examination Survey in the United States population, early life exposure to BPA	Low-grade urinary albumin excretion	Trasande et al., 2013a
	National Health and Nutrition Examination Surveyin the United States population, BPA	Positive association of exposure with the Albumin to creatinine ratio	Kang et al., 2019
	Mice (ICR), Tetrabromobisphenol (TBBPA) prenatal and postnatal exposure, GD0 to PND27	Atrophy of renal tubules and cyst in the kidney	Tada et al., 2006
	Rats (Wistar), Tetrabromobisphenol (TBBPA) exposure, PND4 to PND21	Nephrotoxicity characterized by the formation of polycystic lesions	Fukuda et al., 2004
	Mice (OF1) (pregnant mice exposed to BPA (10 or 100 μg/kg/day), GD9 to GD16	Glomerular abnormalities and changes in glomerular number and density	Nuñez et al., 2018
Phthalates	Taiwan food scandal (2011), early life exposure to di-(2-ethylhexyl) phthalate (DEHP)	Higher micro-albuminuria levels	Tsai et al., 2016; Wu et al., 2018a
Persistent organic pollutants (POPs)	Mice (C57BL/6), 2,3,7,8-Tetrachlorodibenzo-p-dioxin (TCDD), 0.5, 3.0, or 6.0 μg/kg/day, *in utero* and lactational exposure	Effect on renal morphology	Aragon et al., 2008b
	Mice (C57BL/6N), TCDD 6.0 μg/kg/day perinatal exposure	Hydronephrosis and increased renal fibrosis	Aragon et al., 2008a
Heavy metals	Children from a supplementation trial in pregnancy (MINIMat) in rural Bangladesh, arsenic and cadmium	Exposure to cadmium associated inversely with estimated glomerular filtration rate (eGFR)	Skroder et al., 2015
	Antofagasta and the rest of Chile, Arsenic exposure upto 870 μg/L	Increased mortality from cancers and CKD	Smith et al., 2012
	Cross-sectional European survey, Lead (Pb) exposure	Negatively associated with creatinine, cystatin C, and beta2-microglobulin	de Burbure et al., 2006
	Yugoslavian birth-cohort study, Pb contaminated areas	Proteinuria observed in offspring	Factor-Litvak et al., 1999
	Pediatric CKD patients, Pb contaminated areas	High prevalence of elevated Pb levels in pediatric CKD	Filler et al., 2012
	Children aged 12 to 15 years, Area in the vicinity of Pb Smelter	Blood Pb level positively associated with multiple urinary renal injury biomarkers	Bernard et al., 1995; Verberk et al., 1996; Fels et al., 1998
	Cross-sectional study on adolescents aged 12 to 20 years, 1.5 μ/dL of median Pb level in blood	Effect on kidney function (GFR)	Fadrowski et al., 2010
	Rats (Sprague-Dawley), Cadmium chloride (CdCl2) exposure between 2.0 to 2.5 mg/kg on GD8, 10, 12,14	Significant increase of beta 2-microglobulin levels but no effect on metallothionein	Saillenfait et al., 1991
	Rats (Wistar), Cd 1.48 mg/kg/day, GD8-GD20	Structural alterations in fetal renal tissue	Jacobo-Estrada et al., 2016
	Rats (Wistar), Cd 400 mg/ L 3 days per week, 3 weeks throughout gestation	Decreased total volume of kidney, medulla, and proximal and distal tubules	Hamidian et al., 2020
	Rats (Wistar), Cd 5 or 10 ppm during pregnancy and lactation	Relative organ weight of kidney decreased significantly	Luo et al., 2015
	Mice (CD1), Arsenic 85 ppm from GD8-GD18	tumors/lesions initiated by prenatal arsenic in the kidney	Tokar et al., 2012
	Rats (Wistar), NaF (100 mg/L), NaAsO_2_ (50 mg/L) during pregnancy and lactation	Disrupted histopathology and ultrastructure in the kidney with altered creatinine, urea nitrogen and uric acid levels	Tian et al., 2019b
	Rats (Sprague-Dawley), HgCl2 1 mg/kg from GD14-GD21	Increase in urinary beta 2 microglobulin (β2M) and albumin and transient renal dysfunction	Bernard et al., 1992

Detectable bisphenol A levels were reported in a study on children correlated with increased levels of ACR (Trasande et al., 2013a). Furthermore, in some animal studies, gestational exposure to BPA was associated with glomerular abnormalities, including changes in the glomerular number and density (Nuñez et al., 2018). Early-life exposure to BPA and phthalates is also associated with increased tubular injury and oxidative stress, which can affect renal function (Jacobson et al., 2020). In a study on children, exposure to high molecular weight phthalates was associated with higher ACR (Trasande et al., 2014). Exposure to di-(2-ethylhexyl) phthalate (DEHP) tainted food was associated with micro-albuminuria in children (Tsai et al., 2016; Wu et al., 2018a).

Growing evidence suggests a strong association between dioxin exposure and renal dysfunction in adults (Huang et al., 2016), although there is less information on prenatal dioxin exposure and renal abnormalities (Table 3). Perinatal exposure to TCDD can affect renal morphology (Aragon et al., 2008b) and promote hydronephrosis (Aragon et al., 2008a). Similarly, exposure to flame retardants, such as organophosphate esters (OPEs) and tetrabromobisphenol A (TBBPA), was associated with CKD in adult epidemiological studies (Kang et al., 2019). In an *in utero* study, exposure to TBBPA was associated with renal tubule atrophy and cyst in the kidney (Fukuda et al., 2004; Tada et al., 2006).

The heavy metals, which might modulate the endocrine system, are cadmium, arsenic, mercury, lead, manganese, and zinc. However, not all heavy metals are associated with kidney diseases. While there are several adult studies on exposure to heavy metals and nephrotoxicity, the studies on gestational and perinatal exposure are sparse. A cross-sectional study on preschool girls (age range: 4.4–5.4 years) showed that cadmium exposure during childhood was inversely correlated with estimated glomerular filtration rate (eGFR), which can adversely affect kidney function (Skroder et al., 2015). In an animal study, increased levels of albumin, osteopontin, vascular endothelial growth factor, and tissue inhibitor of metalloproteinases-1 were observed in the amniotic fluid of cadmium-exposed mothers (Jacobo-Estrada et al., 2016). In the same study, histopathological assessment of the kidney of the fetus showed tubular damage and precipitations in the renal pelvis. In a related study, gestational and lactational exposure to cadmium led to the reduced relative weight of liver and kidneys in the female offspring (Luo et al., 2015; Hamidian et al., 2020). Gestational exposure to cadmium also caused a significant decrease in the glomerular filtration rate (GFR) with a disorganized expression of tight-junction proteins, such as claudin-2 and claudin-5 in rat offspring (Jacquillet et al., 2007). In another study, rat offspring, which were prenatally exposed to cadmium chloride (CdCl_2_), had increased β2-microglobulin (β2M) levels, suggesting some kidney damage (Saillenfait et al., 1991).

The effect of early exposure to arsenic on the progression and development of kidney diseases has been assessed in few human studies (Smith et al., 2012; Hawkesworth et al., 2013; Zheng et al., 2014; Weidemann et al., 2015). Exposure to arsenic has also been linked with increased mortality in young adults due to multiorgan cancers and chronic renal diseases (Smith et al., 2012). The nephrotoxic effects following prenatal arsenic exposure could also be due to dysregulated autophagy (Tian et al., 2019b) and increased oxidative stress and mitochondrial damage (Tian et al., 2019a,b).

Lead-induced nephropathy in young adults was first reported in children in Queensland, Australia (Nye, 1929). Chronic poisoning with lead (blood lead levels > 60 μg/dL) has been associated with nephropathy in children and adults (Ekong et al., 2006), and was characterized by tubulointerstitial fibrosis, tubular atrophy, glomerular sclerosis, and reduced eGFR (Morgan et al., 1966; Loghman-Adham, 1997). Childhood lead poisoning can also promote hypertension (Moel et al., 1985; Hu, 1991; Fadrowski et al., 2010), prolong partial Fanconi syndrome (Loghman-Adham, 1998), and lead to abnormal renal function (Moel and Sachs, 1992; Filler et al., 2012; Fadrowski et al., 2013). In a prospective Yugoslavian birth-cohort study, high blood pressure and proteinuria were observed in offspring born to mothers living near lead-contaminated environment (Factor-Litvak et al., 1999). This was also associated with increased levels of lead in the blood. Hyperfiltration, which is often linked with albuminuria and a transient increase in GFR, was also associated with early-life lead exposure and may be the cause of kidney injury during adulthood (Khalil-Manesh et al., 1992; Weaver et al., 2003; Ekong et al., 2006; Helal et al., 2012). Early-life exposure to lead was positively associated with serum cystatin C levels (Staessen et al., 2001). However, in another study, early-life exposure to lead was negatively associated with serum creatinine and cystatin C (de Burbure et al., 2006). In another population study on children exposed to lead, lead levels in blood were positively associated with multiple urinary renal injury biomarkers, including retinol-binding protein (Bernard et al., 1995), β2M and Clara cell protein (Fels et al., 1998), and N-acetyl-beta-D-glucosaminidase (NAG) (Verberk et al., 1996).

Exposure to all forms of mercury is nephrotoxic (Zalups and Lash, 1994). In the kidneys, the pars recta segment of the proximal tubule is highly susceptible to mercury. Unfortunately, there are very limited studies on the effect of gestational mercury exposure on the kidneys of the offspring. The accumulation of high mercury levels in the kidney of offspring, following exposure during the gestation period, has been reported (Drasch et al., 1994). Animal studies showed transient renal dysfunction in mothers, as well as the offspring with a significant increase in urinary β2M and albumin levels after mercury exposure (Bernard et al., 1992). An unpublished study from our lab has also associated prenatal methyl mercury exposure with decreased glomerulus numbers in the offspring.

## Prenatal Exposure to EDCS and Cardiovascular Diseases

Cardiovascular diseases (CVD) cause an estimated 17.9 million deaths annually (Wang et al., 2016). To date, the majority of epidemiological and animal studies connecting environmental stressors and CVD have focused on a narrow group of EDCs.

In an *in vivo* study, exposure to ioxynil (IOX) and DES during the embryonic stage led to disrupted cardiovascular development (Li et al., 2019). The study showed increased heartbeat frequency and reduced ventricle volume and an aorta diameter, following IOX and DES exposure during the embryonic stage.

In a similar study, zebrafish embryos, which were exposed to BPA, had impaired cardiogenesis with an altered cardiac phenotype, upregulation of hand 2, estrogen receptor (esr2b), histone acetyltransferase (kat6a), and histone acetylation (Lombó et al., 2019). Furthermore, the increased rate of heart failures in the progeny was observed when male zebrafish was exposed to BPA during spermatogenesis (Lombó et al., 2015). This continued till the F2 generation. The study also showed a significant decrease in five key genes involved in cardiac development in the embryos of the F1 generation (*myh6, cmlc2, atp2a2b, sox2*, and *insrb*). A study on apes, which were orally administered BPA during the gestation period, suggested its impact on the cardiovascular fitness of the developing fetus (Chapalamadugu et al., 2014). A significant decrease in the expression of myosin heavy chain, cardiac isoform alpha (Myh6), was observed in the left ventricle. Similarly, overexpression of ′A Disintegrin and Metalloprotease 12′, long isoform (Adam12-l), was observed in both the ventricles and the right atrium of the heart of the exposed fetus. A study on sheep showed that prenatal BPA exposure followed by postnatal overfeeding leads to a significant increase in interventricular septal thickness and affects the morphological and functional parameters of the heart when the exposed animals become obese later in life (Mohan Kumar et al., 2017).

Animal studies on perinatal BPA exposure have reported enhanced male and reduced female sex specific differences in velocity of the circumferential shortening and ascending aorta velocity time integral. Elevated diastolic blood pressure was observed in all the perinatally exposed female offspring (Cagampang et al., 2012; Patel et al., 2013). Several calcium homeostasis proteins (sarcoendoplasmic reticulum ATPase 2a (SERCA2a), sodium calcium exchanger-1, phospholamban (PLB), phospho-PLB, and calsequestrin 2, which are involved in contraction and relaxation of cardiac muscles were altered (Cagampang et al., 2012; Patel et al., 2013). Fibrosis was also observed in the heart of the fetus prenatally exposed to BPA. This was associated with a significant change in miR-17-5p,−208-3p, and−210-3p expression in the fetal heart (Rasdi et al., 2020). In a study, using PXR-humanized apolipoprotein E-deficient (huPXR∙ApoE^−/−^) mouse model, perinatal exposure to BPA worsened atherosclerosis in adult male huPXR∙ApoE^−/−^ offspring (Sui et al., 2018). However, no significant atherosclerotic changes were observed in female offspring.

In a cross-sectional study, early-life exposure to BPA was associated with elevated diastolic blood pressure (Khalil et al., 2014). In another cross-sectional study on children and adolescents, dietary phthalate (DEHP) exposure was associated with elevated systolic blood pressure (Trasande et al., 2013b). Perinatal exposure to hexachlorobenzene (HCB) was associated with an elevated systolic blood pressure, and early-life exposure to DDE was associated with an increase in diastolic blood pressure (Vafeiadi et al., 2015). A combined cohort study demonstrated that women prenatally exposed to DES are at higher risk of coronary artery disease (CAD), myocardial infarction (MI), high cholesterol levels, hypertension, and elevated blood pressure (Troisi et al., 2013, 2018). All these factors are major signs of metabolic disorders.

In a cross-sectional study on children, exposure to phthalates, such as monobenzyl, monocarboxyoctyl, and monocarboxynonyl during the gestation period, was associated with decreased levels of 8-isoprostane at 9 years of age (Tran et al., 2017). 8-isoprostane is a known marker for oxidative stress, which can promote detrimental metabolic changes later in life. Whereas, at 14 years of age, a positive association (increase) was observed between 8-isoprostane and two other metabolites of high molecular weight phthalates [mono(2-ethylhexyl) and mono (2-ethyl-5-carboxypentyl) phthalate]. A positive association was also observed between 8-isoprostane and total cholesterol levels, as well as systolic and diastolic blood pressure (Tran et al., 2017). Exposure to DEHP during lactation altered the expression of insulin-signaling molecules in the cardiac tissue of the offspring (Mangala Priya et al., 2014). *In utero* exposure to DEHP can also reduce locomotor activity at postnatal day (PND) 60. At later-life stages (PND 200), both systolic and diastolic systemic arterial pressure and locomotor activity were reduced in adult rats perinatally exposed to DEHP (Martinez-Arguelles et al., 2013). Wu and coworkers observed an inverse correlation between early-life exposure to phthalate and systolic blood pressure in boys (Wu et al., 2018b). Another group of researchers identified a notable correlation between different phthalate metabolites such as mono-butyl phthalate (MBP), mono-benzyl phthalate (MBzP), and mono-2-ethylhexyl phthalate (MEHP), and elevated blood pressure in children and adolescents aged 6–18 years (Amin et al., 2018). As per the Spanish INMA-Sabadell Birth Cohort Study, high- and low-molecular weight phthalate metabolites were associated with lower systolic blood pressure in girls but not in boys (Valvi et al., 2015).

Perfluorononanoic acid (PFNA), a perfluoroalkyl substance (PFASs), was associated with elevated systolic blood pressure, low-density lipoprotein cholesterol (LDL-C), and total cholesterol (Khalil et al., 2018). Perfluorooctanoic acid (PFOA) and perfluorooctane sulfonic acid (PFOS) were associated with increased LDL-C. PFOA exposure was also positively correlated with total cholesterol (Khalil et al., 2018). Elevated blood pressure was observed in offspring born to rats exposed to DEX, PFOS, atrazine, and PFNA (Rogers et al., 2014).

Nearly five decades ago, a set of autopsy case reports established an association between perinatal arsenic exposure and cardiovascular conditions, such as myocardial infarction, vascular lesions, and thickening of the arteries in young children (Rosenberg, 1973, 1974). Children with these severe cardiovascular issues had resided in regions of Chile, who were highly contaminated with arsenic (average levels of 870 μg/L) from 1958 to 1970. Young adults (30–49 years) born during this period were at three times higher risk of mortality due to myocardial infarction as compared with the rest of the Chile (Yuan et al., 2007). Several epidemiological studies have also associated *in utero* and early-life arsenic exposure with an increased risk of childhood cardiovascular disease (Table 4). Increased childhood (5–18 years old) mortality due to CVD was observed in arsenic-exposed children from Bangladesh (Rahman et al., 2013). The risk was comparatively higher in girls and in adolescents. According to MINIMat cohort study in Bangladesh, higher *in utero* arsenic exposure was associated with increased blood pressure in children at 18 months of age (Hawkesworth et al., 2013). Elevation in blood pressure from an early age may have detrimental effects later in life, particularly in a genetically susceptible population. In a cross-sectional study of children (3–14 years) in Zimapan, Mexico, a positive correlation was established between total urinary arsenic and carotid intima-media thickness (cIMT), a subclinical indicator of CVD (Osorio-Yanez et al., 2013). The study also showed an association of total urinary arsenic with increased plasma levels of asymmetric dimethylarginine (ADMA), an endogenous inhibitor of nitric oxide production and predictive of CVD. The mothers of the participating children were reported living in the highly contaminated areas during their pregnancy, suggesting a possible contribution of prenatal arsenic exposure in the onset of observed effects. Apolipoprotein E deficient (ApoE^−/−^) mice are highly vulnerable to atherosclerosis. The male offspring of pregnant ApoE^−/−^ mice, which were exposed to arsenic, showed accelerated atherosclerotic plaque and loss of endothelial cell function and vascular tone at 10 and 16 weeks of age (Srivastava et al., 2007). The follow-up study showed a more profound effect, following early postnatal exposure to arsenic (Srivastava et al., 2009).

**Table 4:** EDC exposure and cardiovascular function.

**EDC**	**Study system**	**Key findings**	**Reference**
Bisphenol A (BPA)	Cross-sectional study on Early life exposure to BPA; Obese children 3–8 years old	Increased diastolic blood pressure	Khalil et al., 2014
	Zebrafish, Embryonic exposure, BPA 2 and 4 ppm	Impaired cardiogenesis	Lombó et al., 2019
	Zebrafish, Paternal exposure, BPA 0.1 and 2 ppm	Increased rate of heart failure; abnormal expression of cardiac development genes in offspring born	Lombó et al., 2015
	Rhesus monkeys (Macaca mulatta), gestational exposure, BPA 400 μg/kg	Altered fetal heart transcriptome	Chapalamadugu et al., 2014
	Sheep, Gestational exposure, BPA 500 μg/kg	Interventricular septal thickness in the heart of the offspring	Mohan Kumar et al., 2017
	(Mice 9C57bL/6N), Perinatal exposure, BPA 0.5 and 5.0 μg/kg/day	Velocity of the circumferential shortening and ascending aorta velocity time integral increased in male and decreased in female; increased diastolic blood pressure in females; abnormal expression of proteins involved in contraction and relaxation; increased global methylation in males and reduced in females offspring	Cagampang et al., 2012; Patel et al., 2013
	Rat (Sprague Dawley), Gestational exposure, BPA 0.05 and 0.2 ppm	Heart fibrosis; abnormal expression of miRNAs in offspring	Rasdi et al., 2020
	Mice [PXR-humanized apolipoprotein E-deficient (huPXR∙ApoE^−/−^)], Perinatal exposure, BPA 50 mg/kg	Accelerated atherosclerosis in offspring	Sui et al., 2018
Diethystilbestrol (DES)	Combined cohort study, Women prenatally exposed to DES	Higher risk of CAD, MI, high cholesterol, hypertension and elevated blood pressure	Troisi et al., 2013, 2018
	Zebrafish, Embryonic exposure, DES 0.1 μM	Increase heartbeat frequency, reduced ventricle volume and aorta diameter	Li et al., 2019
persistent organic pollutants (POPs)	Rhea mother-child cohort study, Children 4 years old; Early life exposure to POPs	Increased blood pressure	Vafeiadi et al., 2015
	Cross-sectional pilot study, Children 8–12 years old; Early life exposure to perfluoroalkyl substance (PFAS)	Elevated blood pressure; increased Low-density lipoprotein cholesterol (LDL-C) and total cholesterol	Khalil et al., 2018
Phathalates	Cohort study, Children 6–19 years old; Early life exposure to phthalates	Elevated blood pressure	Amin et al., 2018
	Spanish INMA-Sabadell Birth Cohort Study, Children 4–7 years old; Early life exposure to phthalates	Lower systolic blood pressure z-score in girls	Valvi et al., 2015
	Cross-sectional study, Children 6–19 years old; Early life exposure to di-2-ethylhexyl phthalate (DEHP)	Increase systolic blood pressure	Trasande et al., 2013b
	Cross-sectional study, Children 9–14 years old; Early life exposure to phthalates	Increased total cholesterol, systolic and diastolic blood pressure	Tran et al., 2017
	Rar (Sprague-Dawley), Gestational exposure, DEHP 300 mg/kg/day	Reduced locomotor activity; elevated blood pressure.	Martinez-Arguelles et al., 2013
	Rat, lactational exposure, DEHP 0, 1, 10, and 100 mg/kg /day	Altered expression of insulin signaling molecules in heart tissue of the offspring	Mangala Priya et al., 2014
Heavy metals	Population study; MINIMat cohort study in Bangladesh, Perinatal exposure to arsenic (As)	Increased risk of myocardial infarction (MI), vascular lesions and thickening of the arteries; increased blood pressure; risk comparatively higher in females	Rosenberg, 1973, 1974; Yuan et al., 2007; Hawkesworth et al., 2013; Rahman et al., 2013
	Cross-sectional study, Children 3–14 years old; Early life exposure to As	Increase in carotid intima-media thickness (cIMT), increase blood pressure, greater left ventricular mass and a lower rejection fraction	Osorio-Yanez et al., 2013, 2015
	Mice (Apolipoprotein E deficient), *in utero* exposure, As 85 mg/L daily, GD8 to GD 20	Early onset of atherosclerosis	Srivastava et al., 2007

## Determining Common Mechanisms Associated With Exposure to EDCS

**Figure 1:**
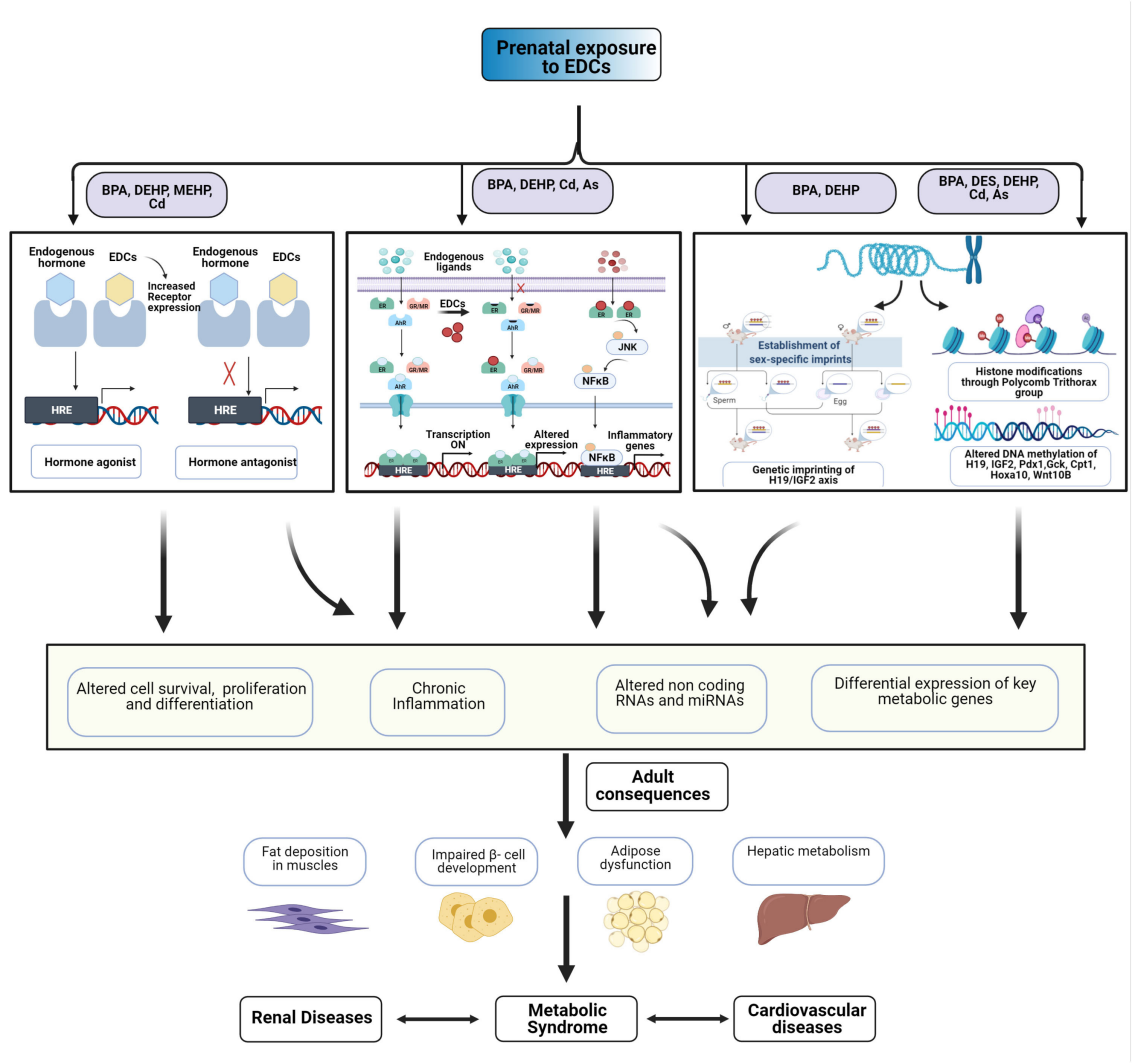
Multiple effects of exposure to endocrine disrupting chemicals (EDCs). EDCs might act through multiple mechanisms to alter cell fate and function. Early-life exposure to EDCs might cause epigenetic dysregulation, pro-inflammatory changes, and changes in energy homeostasis and glucose regulation. These could adversely affect the cardiometabolic renal health of offspring.

EDCs might cause similar modes of action, transport, and storage within tissues and activate or antagonize nuclear hormone receptors (Casals-Casas and Desvergne, 2011; Heindel et al., 2017). During pregnancy, exposure to EDCs has been associated with an abnormal gestational endocrine milieu, including altered levels of sex hormones (Sathyanarayana et al., 2014; Johns et al., 2015). Multiple mechanisms have been proposed for the action of EDCs, including epigenetic modulations, altered inflammatory and oxidative stress responses, hormonal imprinting and fundamental changes in energy storage, and glucose homeostasis pathways. The common mechanisms across multiple classes of EDCs are summarized in Figure 1.

Abnormal maternal environment may alter the developmental trajectory of the fetus via epigenetic modulations (Walker, 2016). Prenatal exposure to phthalates [particularly to DEHP, MEHP, benz-butyl phthalate (BBP), DBP, and MBP] has been shown to modulate genes through their effect on DNA methylation, histone modifications (acetylation, methylation, phosphorylation, ubiquitination, sumoylation, and ADP ribosylation), non-coding RNAs and micro RNAs (miRNAs) (Martinez-Arguelles and Papadopoulos, 2016; Solomon et al., 2017; Dutta et al., 2020). Paternal factors such as sperm DNA changes can also affect fetal development (Day et al., 2016). Prenatal cadmium exposure was associated with hypermethylation in the promoter of glucocorticoid receptor (GR) gene, resulting in increased expression of hepatic GR, leading to dysregulated hepatic metabolism (Castillo et al., 2012).

Several studies link early-life epigenetic changes with dysregulation of metabolic parameters. Early-life exposure to BPA can promote hypermethylation of imprinted genes like IGF2, resulting in impairment of β-cell function in the pancreas of offspring (Mao et al., 2017) and pregnant mothers (Bateman et al., 2017). Maternal exposure to BPA might inhibit the expression of pancreatic and duodenal homeobox-1(Pdx1) gene through deacetylation, demethylation of histone 3 lysine 4 (H3K4), and methylation of histone 3 lysine 9 (H3K9), leading to impaired β-cell development in the offspring (Chang et al., 2016). Maternal BPA exposure also inhibits hepatic glucokinase (Gck) expression through hypermethylation (Maet al. 2013; Liet al., 2014). BPA might exert hyperlipidemic effects through epigenetic modifications of hepatic genes Fasn, Nrf2, and SREBP-1C, which are involved in lipid metabolism (Strakovsky et al., 2015; Shimpi et al., 2017). Similarly, prenatal exposure to DES caused developmental programming of obesity through hypermethylation of the homeobox gene HOXA10 and other region-specific alterations (Bromer et al., 2009). Prenatal BPA exposure could also promote long-term weight gain through its effect on imprinted genes, such as MEST (Junge et al., 2018) and IGF2R (Choi et al., 2020).

Mono-(2-ethylhexyl)phthalate exposure in RAW 264.7 cells was found to cause inflammation through sirtuins (Park et al., 2019a). Phthalates might also induce miRNAs modifications to alter the cholesterol efflux in RAW 264.7 cells (Park et al., 2019b). Maternal exposure to phthalates (DEHP) was also associated with altered DNA methylation in liver (Wen et al., 2020) and hypermethylation of genes (Pdx1, Pax4/6, and HNF-4α) involved in pancreatic β-cell development (Rajesh and Balasubramanian, 2015). All these could promote diabetes and obesity phenotype, which was closely linked with metabolic syndrome.

Non-epigenetic effects on glucose metabolism and adipogenesis have also been observed. Adult exposure to cadmium was found to inhibit key glucose metabolizing enzymes involved in glycolysis, a pentose pathway, gluconeogenesis, and a glycogenesis process through its binding to cysteine-SH residues (Viselina and Luk'Yanova, 2000; Ramírez-Bajo et al., 2014). Cadmium also affects the synthesis, transportation, and metabolism of lipids (Lucia et al., 2010; Yang et al., 2013). Arsenic might also interfere with glucose metabolism through binding with the thiol-containing enzymes (Kannan and Flora, 2004). It can also affect the binding of substrate to insulin receptors and alter adipogenic differentiation and glucose homeostasis (Garciafigueroa et al., 2013; Castriota et al., 2020). Arsenic might also interfere and replace the phosphate group in enzymes involved in glycolysis and oxidative phosphorylation through a process known as “arsenolysis” (Kannan and Flora, 2004).

The promotion of persistent inflammation and oxidative stress is another common theme across multiple EDCs. Cadmium is associated with oxidative stress in the pancreas (Lei et al., 2005). Arsenic can also promote oxidative stress through both mitochondria-dependent (Naranmandura et al., 2011) and independent mechanisms (Kannan and Flora, 2004; Shi et al., 2004; Naranmandura et al., 2011). Upon exposure to arsenic, there could be the production of nitric oxide and superoxide anions, which can subsequently be converted to reactive species to cause oxidative stress and cellular damage through mitochondria-independent mechanisms. Many human and animal studies have demonstrated that EDCs might modulate the immune system and alter the inflammatory cytokine milieu in both the mother and the fetus (Ferguson et al., 2011; Dietert, 2012; Song et al., 2017). Cohort studies on pregnant mothers have demonstrated that detectable levels of paraben and phenols in the urine were associated with abnormal inflammatory cytokine levels in the blood (Watkins et al., 2015; Zota et al., 2018). Aberrant maternal proinflammatory state, in turn, is associated with adverse birth outcomes, including complete loss, preterm labor, preeclampsia, and fetal growth restriction (Cotechini and Graham, 2015; Amaral et al., 2017; Boyle et al., 2017; Catalano and Shankar, 2017).

Several signaling intermediates have been associated with early-life exposure to EDCs and inflammation. Maternal cadmium exposure might activate NF-κB to stimulate the expression of inflammatory cytokines (Ronco et al., 2011). Chronic inflammation after BPA exposure was associated with activation of JNK and NF-kB-signaling pathways that was followed by upregulation of inflammatory cytokines (Savastano et al., 2015; Liao et al., 2016). Exogenous exposure to BPA induced mitochondrial damage in INS-1 cells (Lin et al., 2013; Shirani et al., 2019), along with induction of other proapoptotic proteins (Gong et al., 2017; Wang et al., 2017; Kaur et al., 2018) and cytochrome C release (Hwang et al., 2013).

Many EDCs might modulate physiological processes and hormonal action in a non-monotonic manner (Casals-Casas and Desvergne, 2011; Vandenberg et al., 2012; MacKay and Abizaid, 2018) through their effect on hormone receptors. BPA can promote metabolic disorders through its endocrine-disrupting effect on multiple nuclear receptors, including the estrogen (Ohlstein et al., 2014; Acconcia et al., 2015), glucocorticoid (GR) (Zhang et al., 2019), and aryl hydrocarbon receptor (AhR) (Nishizawa et al., 2005). BPA might act as anestrogen receptor alpha (ERα) agonist through activation of ERα ligand bonding domain (LBD), although it cannot activate the LBD of estrogen receptor beta (ERβ). Thus, it acts as an ERβ antagonist through inhibition of the downstream p38/MAPK pathway (Ascenzi et al., 2006; Bolli et al., 2010). BPA can also enhance adipogenesis through GR activation and translocation in 3T3L1 cells (Sargis et al., 2010; Atlas et al., 2014). Some *in silico* studies also demonstrate its activity on GR (Prasanth et al., 2010; Zhang et al., 2017). Although BPA cannot directly activate the nuclear receptor PPARγ (Ohlstein et al., 2014), its analogs are known to act through activation of PPARγ-RXR complex (Riu et al., 2011). *In utero* exposure to BPA has been also shown to increase the expression of AhR at both RNA and protein levels (Nishizawa et al., 2005). Phthalates are also known to act on hormone-signaling agents to enhance cell proliferation (Jin et al., 2008; Chen et al., 2016). They can either activate the downstream estrogen signaling (Lee et al., 2014) or stimulate AhR and downstream signaling (Hsieh et al., 2012). Adult exposure to phthalates is also reported to cause estrogen receptor stress and autophagy in zebrafish liver through activation of the *IRE-XBP1* pathway (Zhang et al., 2021).

Heavy metals, including arsenic and cadmium, can also alter hormone signaling by interacting with ERα (Stoica et al., 2000) and GR (Simons et al., 1990). Mercury is also known to inhibit the endogenous hormone binding of GR and mineralocorticoid receptor (MR) (Galigniana and Piwien-Pilipuk, 1999; Brkljačić et al., 2004). Arsenic can target the DNA-binding domain (DBD) of GR to inhibit gene expression in rat EDR3 hepatoma cells at higher doses, while the effects are stimulatory at lower doses (Bodwell et al., 2004). Perinatal exposure to arsenic can interfere with MAPK signaling through the reduction in Ras and Raf expression, which are downstream of GR (Martinez-Finley et al., 2011; Caldwell et al., 2015). Chronic exposure to arsenic is known to reduce the circulatory estradiol levels as well as the expression of estrogen receptor and the estrogen-responsive genes (VEGF, cyclin D1, and CDK4) in the uterus (Chatterjee and Chatterji, 2010). Cadmium is also reported to act as a metalloestrogen (Aquino et al., 2012). *In vitro* studies have shown that cadmium could affect the estrogen receptor either through binding to LBD (Stoica et al., 2000) or replacing the Zn^2+^ in DNA binding domain (DBD) of ERα (Low et al., 2002; Nesatyy et al., 2005). *In vivo* studies on the zebrafish brain have also shown that the estrogen receptor antagonistic activity of cadmium can be ameliorated by Zn treatment (Chouchene et al., 2016).

## Conclusion

The clustering of metabolic and cardiovascular risk factors, which was previously known as “syndrome X,” is now referred to as “cardiometabolic syndrome” (CMS). Prenatal exposure to EDCs could cause adverse metabolic alterations, which are closely linked with CKD and CVD. The possible unifying mechanism could involve fundamental changes in the hormonal and epigenetic imprints caused due to EDCs exposure during fetal development. This could permanently alter the baseline inflammatory state and affect adipocyte development in the offspring. All these factors could increase the overall risk for cardiovascular disease, diabetes, and CKD in offspring. Developmental exposure to EDCs may thus be imprinting a risk phenotype for overall cardio-metabolic-renal health (CMR-health) in later life, which needs to be studied further.

## Author Contributions

RS and KK conducted the comprehensive literature review. RS, KK, and VSh wrote the manuscript. RT, HK, and VSh helped in formulation of tables and figure and revised the article for important intellectual content. VSr and VSh designed the concept of the manuscript. All authors read and approved the final manuscript.

## Conflict of Interest

The authors declare that the research was conducted in the absence of any commercial or financial relationships that could be construed as a potential conflict of interest.
